# Coping With Discrimination Among African Americans With Type 2 Diabetes: Factor Structure and Associations With Diabetes Control, Mental Distress, and Psychosocial Resources

**DOI:** 10.5888/pcd21.230189

**Published:** 2024-01-25

**Authors:** Natalie McLaurin, Doonya Tabibi, Tianyu Wang, Taha Alhalimi, H. Matthew Lehrer, Louis Harrison, Hirofumi Tanaka, Mary A. Steinhardt

**Affiliations:** 1 Department of Kinesiology and Health Education, The University of Texas at Austin; 2 Department of Psychiatry, School of Medicine, University of Pittsburgh, Pennsylvania

## Abstract

**Introduction:**

Type 2 diabetes undermines diabetes-related health outcomes among African Americans, who have a disproportionately high incidence of the disease. Experiences of discrimination are common among African Americans and compound diabetes-related stress, exacerbating poor health outcomes. Appropriate use of coping strategies may mitigate the detrimental effect of discrimination on diabetes-related outcomes, but examining associations between coping strategies and health outcomes is needed to inform potential interventions. This study assessed the factor structure of the Coping with Discrimination Scale (CDS) among African American adults with type 2 diabetes and examined associations of CDS subscales with measures of diabetes control, mental distress, and psychosocial resources.

**Methods:**

The CDS was administered primarily through churches to African Americans with type 2 diabetes residing in Austin, Texas, and surrounding areas. Data were collected from August 2020 through April 2023. We conducted principal axis factor analysis of the CDS and determined internal consistency for each factor. We computed bivariate and partial correlations between CDS subscales and indicators of diabetes control (hemoglobin A_1c_, diabetes self-management), mental distress (diabetes distress, perceived stress, depressive symptoms), and psychosocial resources (resilience, social support, self-efficacy).

**Results:**

The 284 African American adults (204 women, 80 men) ranged in age from 23 to 86 years (mean [SD] = 62 [11] y). We identified 4 factors: education/advocacy, internalization, strong response, and detachment. Scores were highest for education/advocacy items and lowest for strong response items. Education/advocacy was associated with higher scores on psychosocial resources, whereas detachment was associated with lower scores. Internalization and strong response were associated with higher mental distress. Strong response was associated with higher hemoglobin A_1c_, and education/advocacy was associated with enhanced diabetes self-management.

**Conclusion:**

We suggest health care professionals create culturally tailored interventions that aid individuals in educating others, advocating for themselves, or recognizing situations outside one’s control and detaching from responsibility, rather than internalizing experiences of discrimination or engaging in strong responses that upon reflection are detrimental to one’s health.

**Summary**

**What is already known on this topic?**

African Americans experience racial discrimination and have a disproportionately high incidence of type 2 diabetes. Chronic stress from racial discrimination and diabetes-related stressors exacerbates poor health outcomes.

**What is added by this report?**

Four factors (education/advocacy, internalization, strong response, and detachment) were significantly associated with key measures of diabetes control, mental distress, and psychosocial resources.

**What are the implications for public health practice?**

Interventions that aim to reduce the incidence of type 2 diabetes among African Americans should include tools for cultivating appropriate coping strategies for addressing racial discrimination.

## Introduction

Diabetes is a major chronic disease that affects more than 37 million US adults and is projected to affect 54.9 million adults nationwide by the year 2030 (1). African Americans are disproportionately affected by the diabetes epidemic, with 12.1% of African Americans having the disease, compared with 7.4% of the non-Hispanic White population (2). Given that complications from diabetes (eg, cardiovascular disease, neuropathy, nephropathy, depression) profoundly affect quality of life and life expectancy (3), it is important to identify factors that contribute to the higher prevalence of diabetes among African Americans.

Exposure to discrimination is a factor that is increasingly recognized as an important social determinant of health (4). Discrimination is associated with an increased risk of type 2 diabetes, hypertension, death (5–7), and depression (8). Historically, African Americans have been negatively affected by interpersonal and institutional discrimination. Despite social and political efforts aimed at reducing the pervasiveness of discrimination, African Americans continue to report more experiences of discrimination than other racial or ethnic groups (9).

Experiences of discrimination are a particularly salient type of stressor that can prompt intense affective, cognitive, and behavioral coping responses (10). African Americans are known to use unique coping strategies toward racial discrimination that are different from coping responses toward ordinary stressors (11). Given the strong interplay between stressors, coping, and overall health (12), use of appropriate coping strategies toward discriminatory experiences may attenuate the negative effect of discrimination on diabetes-related health outcomes among African Americans.

The Coping with Discrimination Scale (CDS) was developed to measure the strategies that various minority groups use to cope with discriminatory experiences (13). The scale was validated among minority samples (ie, racial and ethnic minority college students, members of sexual minority groups), and its 5 subscales (education/advocacy, internalization, drug and alcohol use, resistance, and detachment) demonstrated adequate reliability and construct validity (13,14). However, the CDS has not been administered in a sample of African Americans with diabetes. The objective of our study was to assess the factor structure of the CDS among African American adults with type 2 diabetes. We hypothesized that the CDS would exhibit a similar factor structure as when administered among other minority groups (13,14). Another objective was to examine the associations of the CDS factor structure with diabetes-related health outcomes. Finally, we used an intersectionality approach (15) to explore how these associations differ by sex and socioeconomic status.

## Methods

### Study design and participants

This cross-sectional study used baseline data collected from August 2020 through April 2023 from TX STRIDE (Texas Strength Through Resilience in Diabetes Education), an ongoing clinical trial investigating the effectiveness of a resilience-based diabetes self-management education and support program on type 2 diabetes–related health outcomes among African Americans (16). Participants were recruited through predominantly African American churches in Austin, Texas, and the surrounding areas. Inclusion criteria were being African American, aged 18 years or older, and being diagnosed with type 2 diabetes. Individuals were excluded if they were pregnant or lactating or had medical conditions for which changes in diet or physical activity would be contraindicated. All participants completed a self-report survey packet and provided a blood sample for hemoglobin A_1c_ (HbA_1c_). The study protocol was approved by the institutional review board at The University of Texas at Austin, and all participants provided written informed consent. Participants were compensated $50 for their time.

### Measures

**Coping with discrimination.** The CDS measures how individuals cope with discrimination and includes 5 items from each of the following subscales: education/advocacy, internalization, drugs and alcohol use, resistance, and detachment (13). On the basis of feedback from community leaders who believed that several items asking about drugs or alcohol were repetitive and would reduce participant response, we eliminated 3 of the 5 original items on drug and alcohol use (Appendix Box). Participants were asked the extent to which each strategy described the way they coped with discrimination on a scale ranging from 1 (never like me) to 6 (always like me).

**Diabetes control.** Indicators of diabetes control included HbA_1c_ and diabetes self-management. Blood samples for the measurement of HbA_1c_ were obtained and analyzed according to standard laboratory procedures. For most participants (71%), HbA_1c_ was measured by using a laboratory-based testing device, the DCA Vantage Analyzer (Siemens Healthcare Diagnostics). However, during the early stages of the COVID-19 pandemic (August 2020–July 2021), HbA1c was assessed by using the A1CNow Self Check (PTS Diagnostics), a handheld device primarily marketed for at-home use. We previously established the feasibility and bias of the A1CNow and applied the following correction factor (*y* = 0.665 + 1.003*x*) to each HbA_1c_ value obtained with the A1CNow in our study (17).

We used the 12-item Self Care Inventory–Revised (SCI–R) to assess diabetes self-management in the previous month (18). The SCI–R measures perceived self-management adherence, such as checking blood glucose levels, reading food labels, and keeping clinic appointments on a scale ranging from 1 (never do it) to 5 (always do this as recommended without fail). SCI–R items were averaged and then converted to a 100-point scale (18). The internal consistency of the SCI–R was moderately strong (α = 0.77).

**Mental distress.** Indicators of mental distress included diabetes distress, general perceived stress, and depressive symptoms. We used the 4-item Diabetes Distress Scale (DDS) to measure diabetes care–related stress in the previous month (19). Participants responded on a scale ranging from 1 (not a problem) to 6 (serious problem). Sample items are “Feeling overwhelmed by the demands of living with diabetes” and “Feeling that I am often failing with my diabetes regimen.” The DDS score was calculated as the average of the 4 items. The reliability of the DDS was strong (α = 0.88).

Perceived stress was assessed by using the 10-item Perceived Stress Scale (PSS), which measures the stressfulness of life situations in the previous month (20). Participants responded on a scale ranging from 0 (never) to 4 (very often). Sample items are “How often have you felt difficulties were piling up so high that you could not overcome them?” and “How often have you felt that you were unable to control the important things in your life?” The PSS score was calculated by reverse scoring the 4 positively worded items and then summing all 10 items. The reliability of the PSS was strong (α = 0.86).

Depressive symptoms were assessed by using the 9-item Patient Health Questionnaire (PHQ-9), which measures depressive symptoms experienced (eg, depressed mood, feelings of guilt, worthlessness, restless sleep) in the previous 2 weeks (21). Sample items are “Feeling tired or having little energy,” “Trouble concentrating on things, such as reading the newspaper or watching television,” and “Little interest or pleasure in doing things.” Responses ranged from 0 (not at all) to 3 (nearly every day). The PHQ-9 score was calculated as the sum of the 9 items. The reliability of the PHQ-9 was strong (α = 0.85).

**Psychosocial resources.** Three indicators were used to assess psychosocial resources: resilience, social support, and self-efficacy. Resilience was assessed by using the 6-item Brief Resilience Scale (BRS), which measures the capacity to bounce back or recover from stress (22). Participants responded to sample items such as “I tend to bounce back quickly after hard times” and “It does not take me long to recover from a stressful event.” Responses ranged from 1 (strongly disagree) to 5 (strongly agree). The BRS score was determined by reverse scoring the 3 negatively worded items and then calculating the mean of the 6 items (22). The reliability of the BRS was moderately strong (α = 0.74).

Social support was assessed by using the 12-item Multidimensional Scale of Perceived Social Support (MSPSS), which measures perceived support from family, friends, and significant others (23). Sample items are “My family really tries to help me,” “I can count on my friends when things go wrong,” and “There is a special person who is around when I am in need.” Participants responded on a scale ranging from 1 (very strongly disagree) to 7 (very strongly agree), and the MSPSS score was calculated as the mean of the 12 items. The reliability of the MSPSS was strong (α = 0.96).

Self-efficacy was assessed by using the 10-item modified generalized Self-Efficacy Scale (m-SES) which measures confidence in managing life stressors (24). Participants responded on a scale ranging from 1 (not true at all) to 4 (exactly true) to such items as “I am confident that I could deal efficiently with unexpected events” and “I can solve most problems if I invest the necessary effort.” The m-SES was calculated as the sum of the 10 items. The reliability of the m-SES was strong (α = 0.90).

### Statistical analyses

We conducted a principal axis factor analysis with oblique (Promax) rotation of the CDS with the number of factors set to 5 (13). A threshold of Eigenvalues greater than 1 served as the criterion for factor extraction. Items that had a factor loading greater than 0.30 were retained. All factor loadings were reported, and descriptive names were assigned to each factor. In addition, we conducted a parallel analysis as a validity check to confirm the number of factors within the CDS. We determined the internal consistency coefficients for each factor by using Cronbach α. Bivariate and partial correlation coefficients of CDS subscales with measures of diabetes control, mental distress, and psychosocial resources were evaluated. In exploratory analyses, we used 2-way analysis of variance (ANOVA) to examine whether mean scores on the CDS subscales varied by sex and socioeconomic status (SES). We also used Pearson correlations to explore whether the associations among CDS subscales and measures of diabetes control, mental distress, and psychosocial resources varied between groups stratified by sex and SES. We used Statistical Package for the Social Sciences version 28 (IBM Corp) for all analyses.

## Results

Participants were 284 African American adults (204 women, 80 men) ranging in age from 23 to 86 years (mean [SD] = 62 [11] y). The mean (SD) duration of diabetes diagnoses was 11.0 (8.6) years and mean (SD) HbA_1c_ was 8.1% (1.8%) (Table 1). Most participants were taking oral diabetes medications or noninsulin injectables (61%), were married (45%), and had some college or technical school (48%). Participants predominantly worked full time (43%) or were retired (40%).

**Table 1:** Baseline Characteristics of Participants (N = 284) in TX STRIDE (Texas Strength Through Resilience in Diabetes Education)^a^

Variables	Mean (SD) or n (%)
**Sex**	
Female	204 (72)
Male	80 (28)
**Age, mean (SD), y**	62 (11)
**Body mass index, kg/m^2^**	36.5 (8.4)
**Duration of diabetes, y**	11.0 (8.6)
**HbA_1c_, %**	8.1 (1.8)
**Diabetes medication use**	
Oral medications/non-insulin injectable only	173 (61)
Insulin only	22 (8)
Both	68 (24)
No medication	21 (7)
**Marital status**	
Never married	45 (16)
Married	129 (45)
Separated/divorced	82 (29)
Widowed	28 (10)
**Education level**	
High school/GED or lower	64 (23)
Some college/technical school	135 (48)
Undergraduate degree	52 (18)
Graduate degree	33 (12)
**Employment status**	
Full time	123 (43)
Part time	21 (7)
Unemployed	26 (9)
Retired	114 (40)
**Household income level, $**	
<20,000	39 (14)
20,000–39,999	56 (20)
40,000–59,999	65 (23)
60,000–79,999	58 (21)
≥80,000	60 (22)

Abbreviation: GED, General Educational Development.

a Baseline data were collected from August 2020 through April 2023. TX STRIDE is an ongoing clinical trial investigating the effectiveness of a resilience-based diabetes self-management education and support program on type 2 diabetes-related health outcomes among African Americans (NCT04282395) (16). Participants were recruited through predominantly African American churches in Austin, Texas, and the surrounding areas.

### Factor analyses of the CDS

The initial principal axis factor analysis of the 22-item CDS resulted in 4 factors meeting the threshold of Eigenvalues greater than 1. Thus, we discarded the 5-factor structure and conducted a 4-factor analysis. The scree plot supported a 4-factor solution with Eigenvalues greater than 1 and explained 42% of the total variance. The following 3 items cross-loaded (scored >0.30 on ≥2 factors) and were removed: “I directly challenge the person who offended me,” “It’s hard for me to seek emotional support from other people,” and “I believe I may have triggered the incident.” We conducted a third principal axis factor analysis on the remaining 19 items, which produced a solution accounting for 45% of the explained variance and yielded a 4-factor solution. One item, “I do not talk with others about my feelings,” did not load above 0.30 on any factor and was removed.

We conducted a final principal axis factor analysis on the remaining 18 items, which produced a solution accounting for 46% of the explained variance and yielded a 4-factor solution (Table 2). A scree plot of Eigenvalues indicated an acceptable solution. A concurrent parallel analysis showed agreement for a 4-factor solution of the CDS (Figure). Factor 1, education/advocacy, consisted of 5 items and had strong internal consistency (α = 0.89). Factor 2, internalization, consisted of 3 items and had strong internal consistency (α = 0.84). Factor 3, strong response, consisted of 4 items with adequate internal consistency (α = 0.63). Factor 4, detachment, consisted of 6 items with adequate internal consistency (α = 0.62). Mean participant scores were highest for items on the education/advocacy subscale and lowest for items on the strong response subscale.

**Table 2:** Factor Loadings and Internal Consistency Coefficients for Principal Axis Factor Analysis of the Coping With Discrimination Scale^a^

Item	Factor loading				Mean (SD)
	1	2	3	4	
**Education/advocacy**					
I try to educate people so that they are aware of discrimination.	0.89^b^	0.32	0.14	0.20	3.3 (1.7)
I educate others about the negative impact of discrimination.	0.82^b^	0.35	0.13	0.17	3.5 (1.5)
I help people to be better prepared to deal with discrimination.	0.80^b^	0.39	0.20	0.25	3.4 (1.6)
I try to stop discrimination at the societal level.	0.78^b^	0.41	0.23	0.16	3.1 (1.7)
I educate myself to be better prepared to deal with discrimination.	0.65^b^	0.23	−0.02	0.33	4.0 (1.6)
**Internalization**					
I wonder if I did something to offend others.	0.37	0.91^b^	0.27	0.22	2.5 (1.4)
I wonder if I did something wrong.	0.37	0.79^b^	0.31	0.25	2.2 (1.3)
I wonder if I did something to provoke this incident.	0.37	0.71^b^	0.26	0.13	2.5 (1.4)
**Strong response**					
I use drugs or alcohol to take my mind off things.	0.13	0.22	0.70^b^	0.10	1.2 (0.7)
I use drugs or alcohol to numb my feelings.	0.02	0.23	0.63^b^	0.03	1.1 (0.4)
I get into an argument with the person.	0.13	0.30	0.53^b^	0.08	1.6 (0.9)
I respond by attacking others’ ignorant beliefs.	0.20	0.12	0.48^b^	0.11	1.5 (0.9)
**Detachment**					
I do not think that I caused this event to happen.	0.35	0.12	0.02	0.67^b^	3.0 (1.7)
I do not directly challenge the person.	0.31	0.11	−0.04	0.64^b^	2.8 (1.7)
I try not to fight with the person who offended me.	0.30	0.11	0.07	0.49^b^	2.9 (1.9)
I’ve stopped trying to do anything.	−0.02	0.11	0.10	0.40^b^	2.0 (1.3)
I have no idea what to do.	−0.08	0.18	0.24	0.36^b^	1.9 (1.2)
I do not have anyone to turn to for support.	0	0.10	0.06	0.31^b^	2.1 (1.6)
**Eigenvalue**	4.4	1.7	1.3	1.0	—
**Percentage of common variance**	24.7	9.2	7.0	5.6	—
**Internal consistency reliability (Cronbach α)**	0.89	0.84	0.63	0.62	—

Abbreviation: — , does not apply.

a Baseline data collected from August 2020 through April 2023 from TX STRIDE (Texas Strength Through Resilience in Diabetes Education), an ongoing clinical trial investigating the effectiveness of a resilience-based diabetes self-management education and support program on type 2 diabetes-related health outcomes among African Americans (NCT04282395) (16). Participants were recruited through predominantly African American churches in Austin, Texas, and the surrounding areas.

b Primary factor loading.

**Figure:**
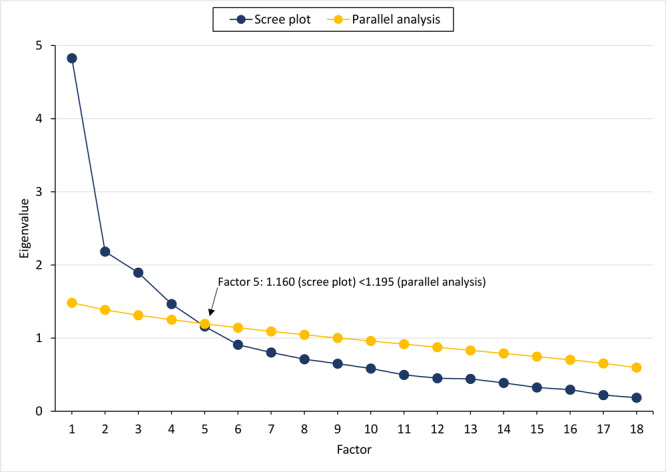
Scree plot of Eigenvalues from the factor analysis and results of the parallel analysis on simulated data with 4 true components underlying 18 variables.

**Table:** 

Factor	Eigenvalue from data	Eigenvalue from parallel analysis
1	4.827	1.483
2	2.183	1.386
3	1.895	1.313
4	1.466	1.253
5	1.160	1.195
6	0.910	1.143
7	0.804	1.092
8	0.712	1.046
9	0.651	1.002
10	0.584	0.961
11	0.497	0.918
12	0.453	0.875
13	0.443	0.832
14	0.387	0.790
15	0.326	0.747
16	0.295	0.704
17	0.221	0.655
18	0.186	0.598

### Association of CDS with diabetes control, mental distress, and psychosocial resources

Bivariate and partial correlation coefficients among the CDS subscales and measures of diabetes control, mental distress, and psychosocial resources were similar. Higher scores on education/advocacy were significantly associated with higher scores on diabetes self-management (*P* = .02) (Table 3). Higher scores on the strong response subscale were significantly associated with higher HbA_1c_ (*P* = .005) and marginally associated with lower scores on diabetes self-management (*P* = .09). Higher scores on internalization and strong response coping strategies were positively associated with higher scores on all 3 indicators of mental distress, including diabetes distress (internalization, *P* = .002; strong response, *P* < .001), perceived stress (internalization, *P* = .003; strong response, *P* < .001), and depressive symptoms (internalization, *P* < .001; strong response, *P* < .001). Higher scores on education/advocacy coping strategies were associated with higher scores on all 3 psychological resources (resilience, *P* < .001; social support, *P* = .007; self-efficacy, *P* < .001) and higher scores on detachment coping strategies were associated with lower scores on all 3 psychological resources (resilience, *P* = .01; social support, *P* = .005; self-efficacy, *P* = .01). Finally, higher scores on strong response coping strategies were marginally associated with lower scores on resilience (*P* = .07).

**Table 3:** Partial Correlations Between Each Subscale of the Coping With Discrimination Scale and Concurrently Measured Study Variables, Controlling for Sex and Household Income^a^

Variable	Mean (SD)	Education/advocacy	Internalization	Strong response	Detachment
**Subscale**					
Education/advocacy	3.4 (1.4)	—	—	—	—
Internalization	2.4 (1.2)	0.41^b^	—	—	—
Strong response	1.3 (0.5)	0.19^b^	0.26^b^	—	—
Detachment	3.1 (0.8)	−0.30^b^	−0.05	0.03	—
**Diabetes control**					
HbA_1c_	8.1 (1.8)	−0.01	0.06	0.17^b^	0.02
Diabetes self-management	54.3 (16.6)	0.14^b^	0.05	−0.10^c^	−0.08
**Mental distress**					
Diabetes distress	2.4 (1.2)	0.02	0.19^b^	0.20^b^	0.01
Perceived stress	14.7 (6.9)	−0.05	0.19^b^	0.24^b^	0.10^c^
Depressive symptoms	5.4 (5.2)	−0.04	0.22^b^	0.25^b^	0.07
**Psychosocial resources**					
Resilience	3.7 (0.7)	0.30^b^	−0.08	−0.11^c^	−0.15^b^
Social support	5.5 (1.3)	0.16^b^	0.07	−0.08	−0.17^b^
Self-efficacy	31.5 (5.0)	0.32^b^	−0.01	−0.06	−0.15^b^

a Baseline data collected from August 2020 through April 2023 from TX STRIDE (Texas Strength Through Resilience in Diabetes Education), an ongoing clinical trial investigating the effectiveness of a resilience-based diabetes self-management education and support program on type 2 diabetes–related health outcomes among African Americans (NCT04282395) (16). Participants were recruited through predominantly African American churches in Austin, Texas, and the surrounding areas.

b *P* < .05.

c *P* < .10.

### Exploratory intersectionality analyses

We found a main effect for sex in which men scored higher on education/advocacy, (*F*_1,274_ = 5.37; *P* = .02) and internalization (*F*_1,275_ = 3.91; *P* = .049) than women. However, the 2-way ANOVAs examining whether mean scores on each of the 4 CDS subscales varied by sex and SES were all nonsignificant (Appendix Supplemental Table 1).

Education/advocacy was associated with higher scores on psychosocial resources for women with low SES (resilience, *P* < .001; social support, *P* = .02; self-efficacy, *P* < .001) and high SES (resilience, *P* = .01; social support, *P* = .04). For women with low SES, internalization was associated with greater mental distress (perceived stress, *P* < .001; depressive symptoms, *P* = .02); strong response was also associated with greater mental distress (diabetes distress, *P* = .002; perceived stress, *P* < .001; depressive symptoms, *P* < .001). Detachment was associated with lower scores on psychological resources (resilience, *P* = .003; social support, *P* = .01; self-efficacy, *P* = .03) (Appendix Supplemental Table 2).

For men with low SES, internalization was associated with greater mental distress (diabetes distress, *P* = .04; perceived stress, *P* = .007; depressive symptoms, *P* = .004), whereas for men with high SES, strong response was associated with greater mental distress (perceived stress, *P* = .02; depressive symptoms, *P* = .002) and lower scores on psychosocial resources (resilience, *P =* .004; social support, *P* = .02; self-efficacy, *P* = .048). Detachment was also associated with greater mental distress (depressive symptoms, *P* = .03) and lower scores on psychosocial resources (social support, *P* = .02; self-efficacy, *P* = .004) (Appendix Supplemental Table 3).

## Discussion

Discrimination is a unique stressor that leads to health inequalities and the persistence of health disparities. Effective coping strategies for dealing with experiences of discrimination are necessary for the livelihood and well-being of minority populations. We found that the CDS has a 4-factor structure (education/advocacy, internalization, strong response, detachment) among African American adults with type 2 diabetes. Higher scores on the strong response subscale were associated with higher HbA_1c_, and higher scores on the strong response and internalization subscales were associated with greater mental distress. Higher scores on the education/advocacy and detachment subscales were positively and negatively associated with psychosocial resources, respectively. Finally, higher scores on education/advocacy were associated with higher scores on diabetes self-management. Collectively, these results suggest that various coping strategies used by African Americans are relevant for diabetes-related health.

The 4-factor structure in our study differs from the 5-factor structure reported by the developers of the CDS, who used a college-aged sample of racial and ethnic minority individuals (13). The subscales education/advocacy and internalization in our study were comparable to those in the original study. However, 2 items originally categorized under drugs and alcohol (“I use drugs or alcohol to take my mind off things” and “I use drugs or alcohol to numb my feelings”) and 2 items originally categorized under resistance (“I get into an argument with the person” and “I respond by attacking others’ ignorant beliefs”) loaded together on a subscale we labeled strong response. One item originally categorized under internalization (“I do not think that I caused this event to happen”) and 2 items originally categorized under resistance (“I do not directly challenge the person” and “I try not to fight with the person who offended me”) loaded on the subscale detachment. The observed differences in factor structure may be due to the differences between the lived experiences of our sample of older African American adults and the experiences of the original sample of college-aged adults (13).

Strong response coping strategies (ie, using drugs or alcohol, getting into an argument, attacking others) in response to chronic exposure to discriminatory experiences may be linked with higher HbA_1c_ through several biobehavioral pathways. Stressors involving social evaluation or uncontrollability (both of which characterize discrimination) activate the hypothalamic–pituitary–adrenal (HPA) axis and trigger the release of cortisol, a stress hormone that also has a role in regulating glucose storage and utilization (27). In a sample of African American adults, elevated cortisol in scalp hair as an indicator of HPA axis function over several months retrospectively was associated with elevated HbA_1c_ independent of demographic factors, chronic health conditions, diabetes medication, exercise habit, and depressive symptoms (28). The broader literature also supports associations among chronic stress, coping, and HbA_1c_. For example, greater use of anger as a coping strategy was associated with higher HbA_1c_ (29), and adaptive emotion-focused coping strategies (ie, changing one’s point of view or mood) were associated with lower HbA_1c_ (30). Additionally, the longitudinal association of negative life events and higher HbA_1c_ has also been observed (31). In our study, in addition to the significant association between the strong response subscale and higher HbA_1c_, strong response also was marginally associated with poorer diabetes self-management. The subscale education/advocacy was also significantly associated with enhanced diabetes self-management. These associations speak to the importance of continued research on the effect of coping with discrimination on diabetes control among African Americans.

The CDS subscales internalization and strong response were both associated with greater mental distress, which is consistent with existing literature. According to the weathering hypothesis, chronic exposure to experiences of social and economic disadvantage are associated with mental health disparities (32). Studies among older African Americans have shown that everyday discrimination is associated with greater depressive symptoms (33) and greater anxiety (34). Future studies should examine a wider range of coping resources to address episodes of racial discrimination and its association with mental health (33). In our study, greater use of internalization and strong response coping strategies — but not education/advocacy and detachment strategies — were associated with greater mental distress. One explanation for this finding is the notion of negativity bias; that is, people tend to focus more on negative emotions and unpleasant stimuli than pleasant stimuli. Negative emotions are often more detrimental to health than the degree to which positive emotions benefit health (35). Future longitudinal research should examine if the negative emotions associated with internalization and strong response coping strategies explain the association between coping strategies and mental health.

It is also plausible that African Americans have learned through experience to respond to discrimination using strategies that protect their mental health (36). Our study supports this postulation, as attempts to educate others or advocate for oneself, or conversely, simply detaching from experiences of discrimination, were associated with less mental distress. Paradoxically, despite the negative effects of discrimination, African American people consistently have better overall mental health than non-Hispanic White people, potentially suggesting a greater ability to bounce back from adversity (37).

Education/advocacy coping strategies were associated with greater psychosocial resources, while detachment coping strategies were associated with fewer psychological resources. Increased psychological resilience and use of support networks can act as protective factors against adversity and are associated with lower levels of inflammation and decreased rates of chronic diseases (38). The use of psychosocial resources may be advantageous for reducing health complications among individuals with diabetes, thus improving their quality of life. While the education/advocacy subscale consists of items in which people directly attempt to educate or end discrimination at the individual and societal level, the detachment subscale consists of items in which individuals distance themselves from the discriminatory event. Detachment may be beneficial for people who experience discrimination, as coping strategies that reflect attempts to withdraw from the stressor are beneficial in situations that are deemed unchangeable (39).

Finally, exploratory findings using an intersectionality approach suggested that associations between the CDS subscales and diabetes outcomes may differ as a function of sex or SES. For example, women with low SES appeared more vulnerable than women with high SES to using more strong response and detachment strategies. Men with low SES appeared more vulnerable to using more internalization strategies than men with high SES, and men with high SES appeared more vulnerable to using strong response and detachment strategies than men with low SES. Although our study included only African American adults, intersectionality of sex and race is important for diabetes-related outcomes (40). Future research should sample participants across spectrums of race, sex, and SES to understand how associations between coping with discrimination and diabetes outcomes may differ by these sociodemographic factors.

### Limitations

Our study has several limitations. First, limitations are inherently associated with 1-time survey data collection, including potentially inaccurate or untruthful responses and common method variance. Second, the study correlations were cross-sectional, and therefore causal inference and directionality cannot be determined. Third, our study asked participants how they cope with experiences of discrimination but did not quantify actual experiences. Future studies should quantify experiences of discrimination and coping strategies. Finally, the results may not generalize to other races or ethnicities or African Americans without diabetes. Nonetheless, examining strategies used by African American adults to cope with discrimination can guide future interventions to prevent discrimination and enhance diabetes-related health outcomes.

### Conclusion

African Americans have a long history of racial discrimination that affects their quality of life and contributes to racial health disparities. The ability of African Americans to cope with discriminatory experiences may attenuate the negative effect of discrimination on diabetes-related health outcomes. Our study provides evidence for a 4-factor structure of the CDS. Education/advocacy coping strategies were more beneficially associated with diabetes-related health, whereas internalization, strong response, and detachment coping strategies had more harmful associations with diabetes-related health. Taken together, our results suggest that different coping strategies toward discrimination are relevant for diabetes-related health among African Americans with type 2 diabetes.

## Appendix Supplemental Materials

**Table:** Box. Original 25 Items of the Coping With Discrimination Scale^a^

Item
**Education/advocacy**
I educate others about the negative impact of discrimination.
I help people to be better prepared to deal with discrimination.
I try to stop discrimination at the societal level.
I try to educate people so that they are aware of discrimination.
I educate myself to be better prepared to deal with discrimination.
**Internalization**
I wonder if I did something to provoke this incident.
I wonder if I did something to offend others.
I wonder if I did something wrong.
I believe I may have triggered the incident.
I do not think that I caused this event to happen.
**Drug and Alcohol Use**
I use drugs or alcohol to take my mind off things.
I do not use alcohol or drugs to help me deal with it.
I use drugs or alcohol to numb my feelings.
I do not use drugs or alcohol to help me forget about discrimination.
I try to stop thinking about it by taking alcohol or drugs.
**Resistance**
I directly challenge the person who offended me.
I get into an argument with the person.
I respond by attacking others' ignorant beliefs.
I try not to fight with the person who offended me.
I do not directly challenge the person.
**Detachment**
It's hard for me to seek emotional support from other people.
I do not talk with others about my feelings.
I do not have anyone to turn to for support.
I've stopped trying to do anything.
I have no idea what to do.

^a^ Source: Wei et al (13).

## Appendix

**Supplemental Table 1:** Results of Independent 2-Way Analysis of Variance Conducted for Each of 4 Subscales of the Coping With Discrimination Scale as Outcome Variables Among African American Women and Men, by SES^a^, Austin, Texas

Variables	Women		Men		Comparison	
	Low SES (n = 122)	High SES (n = 78)	Low SES (n = 38)	High SES (n = 41)	*F*	*P* value
Education/advocacy^b^	3.3 (1.3)	3.4 (1.4)	4.0 (1.2)	3.5 (1.3)	2.79	.10
Internalization^b^	2.3 (1.2)	2.3 (1.2)	2.6 (1.3)	2.6 (1.3)	0.07	.79
Strong response	1.3 (0.5)	1.3 (0.5)	1.3 (0.5)	1.5 (0.7)	0.63	.43
Detachment	3.1 (0.7)	3.1 (0.7)	2.9 (0.9)	2.9 (0.7)	0.43	.51

Abbreviation: SES, socioeconomic status.

^a^ Low SES defined as annual household income <$59,999. High SES defined as annual household income ≥$60,000. In 2019 the median household income for African American adults in Austin, Texas was $46,833 (25). From 2017 to 2021 the median household income in Austin, Texas, was $78,965 (26).

^b^ Main effect of sex, *P* < .05.

**Supplemental Table 2:** Means and Pearson Correlations for Subscales of the Coping With Discrimination Scale and Concurrently Measured Diabetes-Related Outcomes, Among African American Women, by SES, Austin, Texas

Variables	Mean (SD)	Education/ advocacy	Internalization	Strong response	Detachment
**Women: low SES^a^ (n = 122)**					
**Diabetes control**					
HbA_1c_	7.8 (1.8)	0.11	0.12	0.15	−0.11
Diabetes self-management	54.2 (16.0)	0.03	0.03	−0.10	−0.01
**Mental distress**					
Diabetes distress	2.5 (1.2)	−0.06	0.12	0.28^b^	0.04
Perceived stress	15.6 (7.5)	−0.08	0.19^b^	0.30^b^	0.15
Depressive symptoms	5.8 (5.6)	−0.07	0.21^b^	0.35^b^	0.08
**Psychosocial resources**					
Resilience	3.7 (0.7)	0.39^b^	−0.01	−0.08	−0.27^b^
Social support	5.4 (1.4)	0.21^b^	0.01	−0.08	−0.23^b^
Self-efficacy	31.0 (5.7)	0.41^b^	0.05	−0.06	−0.20^b^
**Women: high SES^a^ (n = 78)**					
**Diabetes control**					
HbA_1c_	7.8 (1.6)	−0.06	−0.04	0.09	−0.04
Diabetes self-management	59.2 (13.2)	0.26^b^	0.08	0.05	−0.04
**Mental distress**					
Diabetes distress	2.3 (1.1)	0.10	0.22	0.09	−0.02
Perceived stress	12.9 (5.7)	−0.07	0.05	0.04	−0.14
Depressive symptoms	4.6 (4.4)	−0.08	0.06	−0.02	−0.14
**Psychosocial resources**					
Resilience	3.8 (0.6)	0.28^b^	−0.09	0	0
Social support	5.8 (1.2)	0.24^b^	0.11	0.18	−0.10
Self-efficacy	32.2 (4.2)	0.22	−0.01	0.17	−0.07

Abbreviation: HbA_1c_, hemoglobin A_1c_; SES, socioeconomic status.

^a^ Low SES defined as annual household income <$59,999. High SES defined as annual household income ≥$60,000. In 2019 the median household income for African American adults in Austin, Texas was $46,833 (25). From 2017 to 2021 the median household income in Austin, Texas, was $78,965 (26).

^b^ *P* < .05.

**Supplemental Table 3:** Means and Pearson Correlations for Subscales of the Coping With Discrimination Scale and Concurrently Measured Diabetes-Related Outcomes, Among African American Men, by SES, Austin, Texas

Variable	Mean (SD)	Education/ advocacy	Internalization	Strong response	Detachment
**Men: low SES^a^ (n = 38)**					
**Diabetes control**					
HbA_1c_	8.9 (2.1)	−0.07	0.26	0.20	0.29
Diabetes self-management	49.5 (18.5)	0.26	0.06	−0.04	−0.24
**Mental distress**					
Diabetes distress	2.8 (1.1)	−0.03	0.33^b^	0.26	−0.01
Perceived stress	15.4 (6.9)	−0.07	0.43^b^	0.23	0.24
Depressive symptoms	6.0 (5.3)	−0.10	0.45^b^	0.04	0.16
**Psychosocial resources**					
Resilience	3.9 (0.5)	0.24	−0.26	0.06	0.01
Social support	5.3 (1.4)	0.11	0.06	−0.21	−0.02
Self-efficacy	32.6 (4.4)	0.26	−0.32	−0.09	−0.01
**Men: high SES^a^ (n = 41)**					
**Diabetes control**					
HbA_1c_	8.5 (2.0)	−0.25	−0.17	0.27	0.20
Diabetes self-management	48.2 (19.6)	0.15	0.04	−0.27	−0.20
**Mental distress**					
Diabetes distress	2.3 (1.2)	−0.08	0.05	0.18	0.26
Perceived stress	14.3 (6.8)	0.03	0.15	0.35^b^	0.25
Depressive symptoms	4.8 (5.3)	0.14	0.21	0.47^b^	0.35^b^
**Psychosocial resources**					
Resilience	3.7 (0.5)	−0.01	−0.07	−0.44^b^	−0.29
Social support	5.6 (1.2)	0.16	0.22	−0.37^b^	−0.37^b^
Self-efficacy	31.0 (3.7)	0.19	0.14	−0.32^b^	−0.44^b^

Abbreviation: HbA_1c_, hemoglobin A_1c_; SES, socioeconomic status.

^a^ Low SES defined as annual household income <$59,999. High SES defined as annual household income ≥$60,000. In 2019 the median household income for African American adults in Austin, Texas was $46,833 (25). From 2017 to 2021 the median household income in Austin, Texas, was $78,965 (26).

^b^ *P* < .05.
